# Quantum-Inspired and Non-Classical Approaches to Consciousness: Models, Evidence and Constraints

**DOI:** 10.3390/brainsci16040386

**Published:** 2026-03-31

**Authors:** Oscar Arias-Carrión, Emmanuel Ortega-Robles, Elías Manjarrez

**Affiliations:** 1 División de Neurociencias Clínica, Instituto Nacional de Rehabilitación Luis Guillermo Ibarra Ibarra, Mexico City 14389, Mexico; edortegar@gmail.com; 2 Escuela de Medicina y Ciencias de la Salud, Tecnologico de Monterrey, Mexico City 14380, Mexico; 3 Instituto de Fisiología, Benemérita Universidad Autónoma de Puebla, Puebla 72570, Mexico; elias.manjarrez@correo.buap.mx

**Keywords:** consciousness, quantum neuroscience, zero quantum coherence, neural entanglement, quantum interference, microtubules, Orch OR, quantum neural networks, biophotons, quantum biomarkers

## Abstract

**Highlights:**

**What are the main findings?**

- Quantum formalisms capture structural features of cognition, yet no operational evidence supports the biologically instantiated existence of quantum states in the brain.
- Reported non-classical neural signatures remain preliminary and compatible with classical explanations.

**What are the implications of the main findings?**

- Future research must apply rigorous criteria to distinguish genuine non-classical physical correlations from complex classical dynamics.
- Even without quantum substrates, quantum-theoretic models expand the conceptual tools for studying consciousness.

**Abstract:**

Consciousness presents a structural puzzle: a unified, context-sensitive, globally integrated mode of experience emerging from distributed neural dynamics. While classical neuroscience has mapped synaptic, oscillatory, and network-level mechanisms with increasing precision, debate persists as to whether classical formalisms fully capture the integrative and contextual features of conscious processing. This review examines whether quantum principles offer explanatory leverage in two distinct senses: as formal mathematical frameworks for modeling contextual cognition, and as mechanistic hypotheses proposing biologically instantiated non-classical states. We surveyed empirical and theoretical developments spanning zero-quantum-coherence in MRI signals, entanglement-structured learning paradigms, quantum-inspired computational models, and proposed neural substrates, including microtubules, nuclear spins, and photonic architectures. Although certain findings have been interpreted as consistent with a non-classical structure, no study to date has demonstrated entanglement, long-lived coherence, or collapse dynamics in neural tissue under operational criteria comparable to those used in controlled quantum systems. Replication remains limited, biological entanglement witnesses are not yet established, and nonlinear classical dynamics can reproduce many putative quantum signatures. Accordingly, the decisive question is not whether the brain is quantum, but whether its dynamics exceed the explanatory reach of rigorously defined classical models. Progress hinges on replication, adversarial scrutiny, and operational criteria precise enough to discriminate genuine non-classical correlations from classical complexity. Whether quantum mechanisms ultimately prove necessary or refined classical models remain sufficient, this inquiry compels a deeper understanding of integration, contextuality, and the physical constraints shaping conscious experience.

## 1. Introduction

Understanding how conscious experience arises from neural activity remains one of the most profound challenges in neuroscience. Classical frameworks have elucidated the mechanisms by which neurons communicate, networks oscillate, and cognitive processes unfold, yet these accounts have not resolved how subjective, unified, first-person experience emerges from distributed biological computation. This conceptual gap—articulated in philosophy as the “hard problem” of consciousness [1]—continues to motivate the search for explanatory frameworks that can address the qualitative, global, and context-sensitive character of conscious experience.

Interest has increasingly turned toward whether principles from quantum physics might contribute conceptually or mechanistically to this challenge [2,3]. Several models have been proposed to explain cognition using quantum formalism [4,5,6,7,8,9].

To avoid ambiguity, we briefly clarify the principal quantum concepts referenced throughout this review. Superposition denotes the capacity of a quantum system to exist in a linear combination of multiple possible states simultaneously, rather than occupying a single definite state. Entanglement refers to correlations between subsystems that cannot be decomposed into independent local properties, such that the state of the whole is not reducible to the states of its parts. Decoherence describes the rapid loss of quantum phase relations due to environmental interaction, leading to effectively classical behavior. Measurement in quantum theory involves the transition from a probabilistic state description to a definite outcome under specific interaction conditions. Finally, quantum information formalizes uncertainty and nonclassical correlations using mathematical structures such as Hilbert spaces and noncommutative probability theory. Throughout this manuscript, these terms are used either in their strict physical sense or as formal mathematical analogies, and the distinction is made explicit whenever relevant.

Although the brain operates in a warm, decoherence-prone physiological environment [10,11], research in quantum biology demonstrates that certain quantum effects (for example, coherence or spin-dependent reaction dynamics) can persist under conditions once deemed prohibitive, as in photosynthetic energy transfer and avian magnetoreception [12]. These precedents support plausibility at the level of principle, but they do not establish analogous quantum coherence or entanglement in neural tissue.

Quantum-inspired theories of consciousness span a broad landscape. Some models propose that quantum processes could play an active mechanistic role in neural computation, invoking substrates such as microtubules [13], myelinated axons [14,15], or nuclear spin networks as candidate loci for quantum-relevant degrees of freedom. These proposals vary widely in evidential maturity and should be treated as hypotheses that require direct biophysical validation.

Others employ quantum theory as a formalism rather than a mechanism, using quantum probability or quantum information frameworks to model the non-commutative, context-dependent, and globally integrated nature of cognition [7,8]. Across these perspectives, the shared premise is that classical descriptions of neural dynamics may be incomplete for certain features of cognition and experience, particularly unity, intentionality, and phenomenological immediacy. Whether this reflects limits of current classical models, the utility of quantum formalisms as descriptive tools, or genuinely non-classical physical mechanisms remains unresolved.

Emerging tools—including modified magnetic resonance sequences, quantum-optical detection methods, and entanglement-sensitive analytical frameworks—have begun to enable more explicit, falsifiable tests of quantum-informed hypotheses [16]. However, the mere availability of quantum-adjacent measurement methods does not by itself establish that any observed effect is quantum in origin.

Early findings have generated considerable excitement but also debate. Distinguishing genuine quantum signatures from artefacts of nonlinear classical dynamics remains a central methodological challenge in the field, as analytical approaches can be sensitive to non-stationarity, multifractality, and chaotic dynamics.

The present review aims to clarify the conceptual foundations, technological landscape, and empirical status of quantum neuroscience. Rather than presupposing that consciousness requires quantum mechanisms, we ask a more neutral and tractable question: Can quantum principles—mechanistic, informational, or formal—offer explanatory value for understanding conscious experience? By separating speculative claims from testable hypotheses and emphasizing the methodological constraints that shape ongoing research, we provide a rigorous and interdisciplinary framework for advancing this emerging domain.

Because “quantum” is used in both mechanistic and formal senses, we explicitly distinguish quantum theory as a mathematical framework for modeling contextual probabilistic cognition and claims that biologically instantiated quantum states (coherence, entanglement, or collapse dynamics) causally contribute to neural computation. Throughout, we grade claims by evidential strength and treat mechanistic quantum hypotheses as provisional until supported by replication, rigorous null models, and operational criteria that can discriminate non-classical correlations from complex classical dynamics.

## 2. Search Strategy

We conducted a structured, semi-systematic literature search across PubMed, Scopus, and Google Scholar covering the period 1990–2025. Our objective was to identify all available scientific work examining potential intersections between quantum mechanics, neurobiology, cognitive neuroscience, and the mechanistic study of consciousness. The search was designed to capture empirical data, biologically grounded hypotheses, and methodological innovations capable of detecting coherence-, entanglement-, or contextuality-related signatures in neural systems.

The search strategy combined broad conceptual terminology with technical domain-specific descriptors. Key search terms included “quantum consciousness”, “quantum brain”, “neural entanglement”, “quantum biology”, “zero quantum coherence”, “orchestrated objective reduction”, “microtubules”, “quantum cognition”, “wavefunction collapse”, “von Neumann entropy”, and “quantum neural networks.” Boolean expansions were tailored to each database, ensuring sensitivity to both classical neuroscience work that metaphorically invokes quantum principles and quantum physics work that proposes plausible biological implementations.

Eligible studies met at least one of the following criteria: (1) Empirical investigations directly testing hypotheses about quantum-like or non-classical neural dynamics, including MRI protocols adapted for quantum coherence, entanglement-structured EEG paradigms, and behavioral signatures reported to be difficult to explain under standard classical conditioning assumptions; (2) Theoretical or biophysical models proposing mechanistically explicit pathways through which coherence, entanglement, stochastic collapse, or quantum contextuality could influence cognition; (3) Methodological papers describing instruments or analytical approaches with explicit sensitivity to coherence or entanglement (e.g., quantum-adaptive MRI, spin-based magnetometry, entanglement witnesses, QNN-based cognitive modelling).

We excluded studies lacking methodological transparency, biological plausibility, or testable predictions. Additional records were identified through citation chaining, manual screening of seminal papers, and review of experimental datasets that examined anomalous neural signatures—including zero-quantum coherence bursts reported in MRI time series, EEG variance patterns reported under quantum-randomized or entanglement-structured stimulus contingencies, and stochastic-collapse formalisms.

This search yielded a coherent body of literature up to the present date, spanning electrophysiology, neuroimaging, quantum biology, stochastic quantum dynamics, and quantum-inspired computational modelling. Collectively, these studies reveal an emerging pattern: several independent research programs. reveal an emerging pattern: several independent research programs report findings interpreted by their authors as potentially non-classical, though many remain compatible with alternative classical explanations and require stringent replication and adversarial controls.

Although the evidence remains preliminary and often contentious, it supports a cautious working conclusion: the investigation of consciousness may benefit from tools and theoretical models that extend beyond traditional neurobiological assumptions, provided that claims are explicitly graded by evidential strength and tested against well-specified classical null models.

This synthesis forms the evidential foundation for the present mini-review, which aims to evaluate the plausibility, constraints, and future testability of quantum-informed theories of consciousness.

## 3. Theoretical Models of Quantum Consciousness

Several models have been proposed to explain cognition using quantum formalism as a mathematical and probabilistic framework, rather than as a biological substrate (Table 1) [4,5,7,8]. Efforts to integrate quantum mechanics into theories of consciousness require a precise understanding of the foundational principles governing non-classical systems. Although developed to describe the behavior of subatomic particles, these principles—superposition, entanglement, measurement, decoherence, and quantum information—offer a formal vocabulary for describing phenomena that are global, contextual, and probabilistic in ways reminiscent of cognitive and perceptual experience. The central challenge is not to assert that the brain is a quantum computer, but to ask whether quantum frameworks illuminate aspects of consciousness that classical models fundamentally miss.

## 4. The Technological Landscape for Investigating Quantum Brain Processes

The empirical study of quantum phenomena in the brain has reached a stage where specific hypotheses can be operationalized more directly than in past decades. What began as a speculative frontier too bold for classical neuroscience and too complex for traditional physics has become an increasingly test-driven, though still high-uncertainty, research program shaped by rapid innovation.

As tools from quantum optics, condensed-matter physics, and neurobiology converge, the central aspiration is no longer merely philosophical: it is to determine, with empirical precision, whether the brain hosts forms of coherence, entanglement, or contextuality that transcend classical dynamics. Achieving this will require measurement technologies capable of probing processes at the edge of physical law, while embedded in one of nature’s most complex living systems.

Classical methods such as EEG, MEG, and fMRI have underpinned consciousness research for decades. They index macroscopic neural activity with high spatial or temporal resolution yet remain blind to the microscopic correlates of quantum structure. Nonetheless, these tools are undergoing conceptual reinvention. The Zero Quantum Coherence (ZQC) MRI protocol exemplifies this shift. By employing sequence designs that suppress dominant single-quantum nuclear magnetic resonance pathways, ZQC-MRI isolates a residual, sequence-dependent signal component that some authors have interpreted in terms of zero-quantum coherence. Reports describe heartbeat-synchronized signal fluctuations during wakefulness and reductions during decreased arousal (e.g., sleep onset in a subset of participants) [17]. While these findings have been proposed as consistent with dipolar spin interactions or coherence-sensitive dynamics, alternative explanations—such as sequence-specific contrast mechanisms, motion- or flow-related effects, and other physiological confounds—remain plausible. Accordingly, the observations should be regarded as suggestive but not yet definitive evidence of non-classical processes in vivo.

Nevertheless, caution is warranted: vascular artefacts, anisotropic diffusion, and hemodynamic coupling remain plausible confounds. Decisive evaluation will require multi-site replication, blinded acquisition, physiological perturbation, and rigorous control modelling. If validated, ZQC-MRI would represent a novel MRI-based approach sensitive to coherence-related signal components.

At molecular and mesoscopic scales, insights from quantum biology are reshaping expectations. Photosynthetic complexes and avian magnetoreception reveal that biological organisms can maintain functionally relevant quantum effects under ambient thermal conditions, a finding once thought impossible. Inspired by these precedents, theorists have proposed that phosphorus nuclear spins residing in phosphate groups within neural environments could form relatively long-lived spin states under specific biochemical conditions, in principle enabling qubit-like behavior [13,18]. These cases motivate exploration, but they do not directly provide valid demonstrations of mechanistic claims about quantum entanglement in the brain.

Testing this hypothesis will require adapting entanglement-sensitive spectroscopy and spin-resolved magnetometry—technologies well established in solid-state physics but yet to be applied in the living brain.

A further frontier suggests that the nervous system may act as a photonic quantum architecture. Models of myelinated axons propose that their layered dielectric structure forms cylindrical wave-guides that could, in principle, support low-loss photonic propagation and other optical degrees of freedom relevant to quantum-inspired hypotheses [14,15]. If correct, the brain may compute not only with ions and chemicals, but also with photons and fields. Validating this framework will require integrating advanced quantum-optical methods, including single-photon counting, entanglement witnesses, high-efficiency avalanche photodiodes, and only if robust photonic signals are established in vivo, progressively stronger tests of non-classical correlations.

These methods are technologically demanding, but their successful adaptation would enable the first direct tests of quantum photonics in neural tissue.

At the systems level, behavioral and electrophysiological paradigms have begun to operationalise quantum-theoretic predictions. EEG studies using quantum-randomized or entanglement-structured stimulus generation show anticipatory activity patterns and behavioral signatures that some authors argue are not readily captured by standard classical conditioning models [19,20]. The development of the Quantum-Multilinear Integrated Coefficient (Q) [21] or the Posner model [22] provides a mathematically principled measure for quantifying variance aligned with entanglement-structured task design.

Importantly, these paradigms incorporate biomarkers of neuroplasticity—BDNF, salivary alpha-amylase—within preregistered designs, reflecting the methodological maturation of the field. Even so, independent replication and adversarial testing remain essential to probe robustness against expectancy effects, sensory leakage, analytic flexibility, and other hidden confounds.

Perhaps the most transformative innovations arise from hybrid neuro-quantum platforms. Nitrogen-vacancy (NV)-diamond magnetometry, capable of resolving single-spin dynamics, is being engineered for biological compatibility. In combination with optogenetic stimulation, high-resolution calcium imaging, and neural organoids, these systems may allow causal interrogation of whether induced neural states modulate quantum-sensitive signals [16]. Should such platforms succeed, they would establish an unprecedented experimental infrastructure—one capable of testing not only whether the brain sustains quantum processes, but whether these processes interact with cognition in law-like, manipulable ways.

Simultaneously, quantum-inspired computational frameworks have begun yielding insights unattainable with classical models. Quantum neural networks and tensor-network architectures emulate contextuality, interference, and superposition-like representational richness [8,23]. These models do not presume that the brain implements physical qubits; rather, they demonstrate that quantum formalisms provide compact, expressive tools for modelling cognitive operations that resist classical reduction. Their predictive accuracy across decision-making, uncertainty, and inference provides an independent line of support for the relevance of quantum-like structure in cognition.

Nevertheless, substantial methodological challenges remain. Analytical approaches such as multifractal detrended fluctuation analysis (MF-DFA) and cross-recurrence quantification analysis (CRQA), which are frequently deployed to detect putative quantum signatures, are exquisitely sensitive to chaos, multiscale feedback, and fractal structure. Without stringent null models, permutation controls, and transparent preregistration, classical complexity can be easily misinterpreted as quantum coherence [20,24]. The epistemic credibility of quantum neuroscience depends on its ability to distinguish classical high-dimensionality from genuine quantum structure—a problem that demands methodological humility and mathematical sophistication.

In summary, the technological landscape for investigating quantum brain processes is advancing at a pace unthinkable a decade ago. Classical neuroimaging continues to map the correlates of consciousness but lacks quantum specificity. In contrast, quantum sensors, entanglement-aware spectroscopy, spin physics, and photonic detection provide unprecedented sensitivity—yet require bioengineering innovation and interpretive discipline. The future of the field will require interdisciplinary integration: physicists who understand biology, neuroscientists who speak the language of quantum, and computational theorists capable of bridging both worlds. Whether quantum mechanics plays a mechanistic role in conscious experience or serves primarily as a powerful mathematical language remains unresolved. However, for the first time, we possess the conceptual maturity and technological ambition to pose the question scientifically—and, potentially, to answer it.

Additionally, Abramov et al. [25] demonstrated that q-statistics derived from EEG event-interval distributions can differentiate global from local neural complexity, with functional states selectively modulating local complexity at specific scalp sites. The positive correlation between the q parameter and theta power during rich associative states (e.g., music listening) illustrates that complexity measures from non-extensive statistical mechanics can complement quantum-sensitive instrumentation in probing brain organization. However, these measures remain classical in nature.

## 5. Basic Principles of Quantum Mechanics for Consciousness

**Figure 1:**
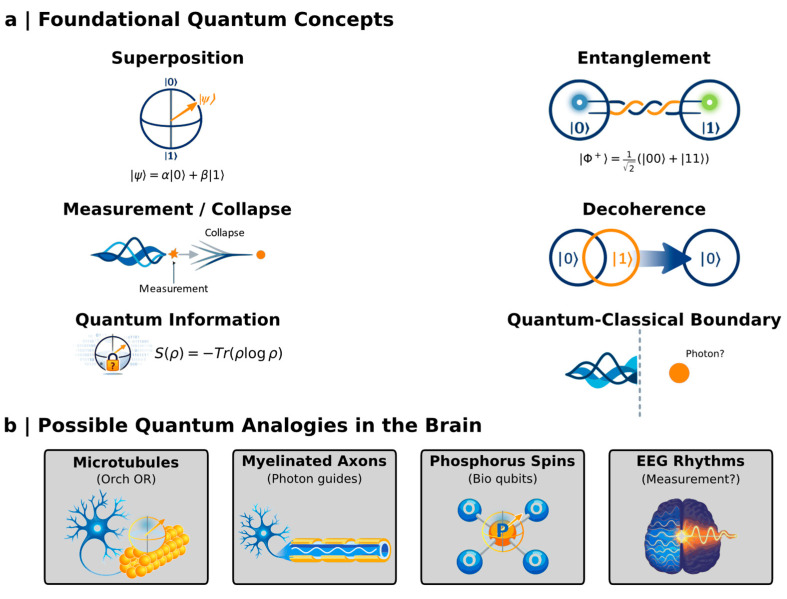
Foundational quantum principles and proposed neural analogies in quantum models of consciousness. (**a**) Foundational quantum concepts. Superposition allows a quantum system to be described as a linear combination of basis states, illustrated by a qubit in the state |ψ⟩ = α|0⟩ + β|1⟩. Entanglement gives rise to correlations between subsystems that cannot be accounted for by local classical variables, exemplified by the Bell state |Φ^+^⟩ = (1/√2)(|00⟩ + |11⟩). Upon measurement, definite outcomes are observed; depending on the interpretation, this is formally represented as a state update or an effective collapse. Decoherence refers to the rapid suppression of phase coherence due to environmental interaction, leading to effectively classical behavior. Quantum information can be quantified using the von Neumann entropy, S(ρ) = −Tr(ρ log ρ), which measures the uncertainty of a quantum state. Together, these principles inform discussions of the quantum–classical transition and highlight the biophysical challenge of sustaining coherence in warm, noisy systems such as the brain. (**b**) Proposed neural analogies. Various biological structures have been hypothesized as potential substrates for quantum-relevant dynamics. Microtubules, central to the Orch OR proposal, have been suggested to participate in orchestrated reduction-like processes, although direct empirical support remains limited. Myelinated axons have been proposed as possible optical waveguides for biophotons, a hypothesis that remains experimentally unsettled. Phosphorus nuclear spins have been discussed as candidate qubit-like degrees of freedom with potentially longer coherence times under certain conditions. At larger scales, some authors have drawn analogies between global EEG transitions and coherence-to-classical shifts, though such interpretations remain speculative. These proposals outline hypothetical pathways through which quantum principles might, in theory, contribute to neural integration and conscious experience.

To consider whether consciousness may draw upon quantum principles is not to abandon neuroscience—it is to expand its conceptual perimeter. Quantum mechanics introduces structural features of reality that have no classical analogue: superposition, entanglement, contextuality, measurement-driven state reduction, decoherence, and quantum information. These principles were never designed for the brain. However, they describe modes of organization—holistic, probabilistic, globally constrained—that echo the very properties of conscious experience classical models struggle to explain (Figure 1). Understanding these principles is therefore not an indulgence; it is a prerequisite for any scientific attempt to determine whether the brain leverages dynamics unavailable to classical physics.

### 5.1. Superposition: Structured Ambiguity and Parallel Representations

Classical systems occupy one state at a time. Quantum systems, by contrast, inhabit superpositions of states, enabling them to encode parallel possibilities within a single physical structure:

(1) $$|\psi \rangle =\alpha |0\rangle +\beta |1\rangle ,|\alpha {|}^{2}+|\beta {|}^{2}=1.$$

Superposition has been advanced as a conceptual analogue for perceptual ambiguity, predictive processing, and the brain’s capacity to entertain multiple competing hypotheses prior to conscious resolution. While no direct evidence yet shows superposition in neural substrates, the mathematical structure of superposition provides a potent model for the preconscious richness underlying decision-making and perceptual inference.

Neven et al. [16] sharpen this idea by conjecturing that conscious experience arises specifically during the formation and dynamical evolution of a quantum-mechanical superposition, rather than at a putative collapse event. They suggest that this shift may circumvent concerns about compatibility with relativistic causality that have been raised in some formulations of objective-reduction models, including aspects of Penrose’s original proposal. On this account, the informational structure of the evolving superposition is taken to determine the qualitative character of experience. At the same time, individual Feynman paths in Hilbert space are interpreted as representing distinct potential sequences of conscious moments within the overall superposed state. While the conjecture remains untested, the authors outline a concrete experimental program, including xenon-isotope anesthetic studies on brain organoids and Drosophila, to probe whether nuclear-spin-dependent superposition effects modulate consciousness.

### 5.2. Entanglement: Beyond Classical Correlation

Entanglement generates correlations between components of a system that cannot be decomposed into independent parts. The Bell state,

(2) $$|\Phi^{+}\rangle =\frac{1}{\sqrt{2}}(|00\rangle +|11\rangle ),$$

Exemplifies such non-local structure. In theoretical neuroscience, entanglement has been invoked to explain global synchrony, rapid integration across distributed networks, and the unified nature of conscious scenes. Regardless of whether entanglement exists physically in the brain, its formal properties map closely onto cognitive features that resist classical modelling—particularly the binding of sensory, affective, and intentional content into a coherent first-person perspective.

Neven et al. [16] further propose that quantum entanglement naturally resolves the binding problem and the unity of phenomenal experience without additional assumptions, with the number of entangled degrees of freedom bounding the content and richness of consciousness. They suggest that brain–quantum computer interfaces mediating entanglement could, in principle, expand conscious experience into higher-dimensional Hilbert spaces, though such technology remains far beyond current capabilities.

### 5.3. Measurement and Collapse: From Potentiality to Experience

Quantum systems evolve smoothly under the Schrödinger equation,

(3) $$i\hbar \frac{d}{dt}|\psi (t)\rangle =\hat{H}|\psi (t)\rangle .$$

When measured, definite outcomes emerge, a process whose formal description depends on the interpretive framework adopted. This apparent dual structure—unitary evolution alongside the emergence of definite outcomes in measurement—has inspired models of consciousness that treat experiential moments as analogous to measurement-like events. The orchestrated objective reduction (Orch OR) framework extends this analogy, proposing that conscious moments correspond to objective reductions driven by gravitational constraints. While controversial, such proposals highlight the need for mechanistic accounts that reconcile continuous neural dynamics with discrete phenomenological moments.

Georgiev [26] argues that collapse-based functionalist models, including Orch OR, render consciousness a causally impotent epiphenomenon, a product of objective reductions rather than their agent. His quantum reductive alternative identifies first-person conscious states directly with unobservable quantum state vectors, proposing that the observable brain is a third-person construct generated when the environment measures a subset of commuting quantum brain observables. While this framework remains speculative and has not been empirically tested, it illustrates how quantum information theory may offer formal tools for addressing the hard problem without invoking emergence.

Similarly, Schiffer [27] contends that quantum collapse in microtubules alone, as proposed by Orch OR, is insufficient to account for subjective experience and instead hypothesizes that consciousness arises from symmetry-breaking interactions between structured neural coherence patterns and fundamental quantum fields. This proposal, while providing testable predictions via biophoton coherence spectroscopy, remains speculative pending empirical confirmation.

Marshall [28] proposes a “Cognitive Interpretation of Quantum Mechanics” in which all living cells act as quantum observers, hypothesizing that the same principle that enables wave-function collapse grants biological organisms agency. Drawing on Turing’s halting problem, he argues that cognition, defined as inductive reasoning, cannot be reduced to computation, and that wave collapse by cellular observers is the physical mechanism for generating negentropy. While this framework remains largely theoretical and awaits direct experimental demonstration, it offers a provocative link between basal cognition research and the measurement problem in quantum physics.

### 5.4. Decoherence: The Thermodynamic Objection and the Biological Counterexample

Decoherence—the rapid loss of quantum coherence through environmental interaction—remains the most formidable challenge to quantum theories of consciousness. The brain’s warm (~310 K), wet, and electrically noisy conditions appear inhospitable to delicate quantum states. Nevertheless, quantum biology has overturned similar assumptions. Photosynthetic complexes maintain coherence for surprisingly long durations, and entangled radical pairs enable avian magnetoreception under ambient thermal conditions. These findings do not confirm neural coherence, but they undermine the argument that biological systems are necessarily incompatible with quantum phenomena.

In support of the view that quantum effects are operationally relevant in biology, Marshall [28] draws on discussions of photosynthetic energy transfer and enzyme catalysis, citing reports that energy-transfer efficiencies in certain light-harvesting systems can approach near-unity and invoking coherent excitonic dynamics as a possible contributing factor. Following Davies, he characterizes such systems as effectively sampling multiple pathways within a quantum superposition before yielding a classical outcome, metaphorically describing this as a biological “Maxwell’s demon.” He interprets these phenomena as illustrative of how organisms might harness quantum dynamics in the service of adaptive function. However, his extension of domain-specific quantum effects in molecular systems to a generalised cellular “observer” mechanism remains speculative and lacks direct empirical evidence for organism-level wave-function collapse.

### 5.5. Quantum Information: Entropy, Uncertainty, and Cognitive Transitions

Quantum systems are not merely physical—they are informational. Von Neumann entropy,

(4) S(ρ)=−Tr(ρlog ρ),

quantifies the uncertainty encoded in a quantum state. Some theorists have proposed that transitions from preconscious processing to conscious awareness may correspond to reductions in quantum informational entropy. While speculative, this view frames consciousness not as a by-product of neural firing but as an informational transition governed by constraints on uncertainty, integration, and representational collapse.

### 5.6. The Quantum–Classical Boundary: Proposed Neural Substrates

Several neurobiological structures have been proposed as potential hosts of quantum-relevant dynamics. Microtubules have been posited to support Orch OR processes via orchestrated coherence and gravitationally induced collapse. Myelinated axons, with their layered dielectric structure, may function as cylindrical resonators that guide entangled biophotons. Phosphorus nuclear spins offer yet another candidate substrate, potentially forming long-lived entangled networks within neuronal microenvironments. None of these proposals is empirically confirmed—but each provides a testable hypothesis about where the quantum–classical boundary might be negotiated within the brain.

The principles of quantum mechanics offer a conceptual and mathematical lens through which consciousness can be examined with renewed precision. They do not, on their own, imply that the brain operates quantum mechanically. Instead, they expand the theoretical horizon, offering new frameworks for interpreting the unity, contextuality, and informational structure of conscious experience. Whether quantum processes serve as metaphors, mechanisms, or both remains an open question, one that only rigorous empirical investigation can resolve.

## 6. Insights from Quantum Computing and Neural Networks

Classical computational models—especially artificial neural networks (ANNs)—have shaped modern theories of cognition by demonstrating how relatively simple computational units can give rise to perception, memory, and decision-making. Nevertheless, despite their success, ANN architectures reveal intrinsic limitations when confronted with the cognitive domains where biological systems exhibit their most extraordinary sophistication: the resolution of ambiguity, the flexible incorporation of context, and the navigation of combinatorial complexity. These constraints have prompted a growing exploration of quantum computation, not as a repudiation of classical models, but as an expansion of our conceptual and mathematical repertoire for understanding mind and brain.

A formal application of Hilbert-space modeling to decision behavior is provided by Yu and Jayakrishnan [29], who developed a quantum cognition model to bridge stated and revealed preferences in travel behavior. In their framework, survey responses and real-world choices are treated as distinct observables acting on a shared mental state vector, with discrepancies interpreted as basis rotations or measurement-induced state changes. The model captures framing effects, sequencing effects, and SP-RP gaps through interference terms derived from quantum probability theory. Importantly, the authors adopt a utilitarian and formal stance: the approach does not posit quantum neurophysiology but instead employs quantum logic as a generalized probabilistic framework for modelling context- and measurement-sensitive cognition. While mathematically elegant, the model’s explanatory value depends on empirical calibration and careful interpretation, as Hilbert-space structure in behavior does not imply the presence of physical quantum processes in neural tissue.

Recent work has explored integrating quantum optimization algorithms into established cognitive architectures. For example, Pathak et al. [30] proposed embedding the Quantum Approximate Optimization Algorithm (QAOA) into the procedural module of the adaptive control of thought-rationale (ACT-R) architecture to enhance combinatorial decision-making performance. In this framework, production rules are mapped onto quantum states, and variational quantum circuits are used to optimize action selection before reintegration into the classical control cycle. Importantly, this approach does not posit quantum physical processes in biological neural tissue; rather, it employs quantum computational methods as algorithmic tools to improve artificial decision systems. While such hybrid architectures illustrate the potential of quantum optimization for structured reasoning tasks, their relevance to biological cognition remains indirect. Demonstrations of improved computational efficiency in artificial systems should therefore be interpreted as advances in algorithmic design rather than evidence for non-classical dynamics in the human brain.

Another recent development in quantum-like cognition is the coupling of neuronal network models with generalized probability theory (GPT). Khrennikov et al. [6] propose embedding weighted directed graphs representing communicating neurons into an operational measurement framework based on ordered linear spaces. In this approach, neuronal signaling is modeled using classical random variables, while cognitive phenomena such as order effects, interference, and non-repeatability arise from the calculus of measurement instruments rather than from quantum state spaces. Importantly, the authors explicitly distinguish this quantum-like formalism from genuine quantum-physical processes in the brain. The state space in their primary model is classical (a convex polytope), and non-classical cognitive effects emerge from non-commutative instrument dynamics rather than from physical superposition or entanglement.

While this framework offers a mathematically rigorous bridge between neural network representations and quantum-like cognitive modeling, it should not be interpreted as evidence for quantum states in biological tissue. Instead, it provides a formal operational structure capable of reproducing non-classical probability effects within entirely classical neuronal dynamics. Its significance lies in clarifying that cognitive non-classicality may arise at the level of probabilistic representation and measurement theory, without requiring microscopic quantum coherence in the brain.

### 6.1. Quantum Neural Networks: Computation in a Higher Algebra

Quantum neural networks (QNNs) incorporate the principles of superposition, entanglement, and interference into computational architectures designed to capture forms of reasoning and adaptation that defy classical formalisms [23]. Unlike classical neurons, which encode discrete values, qubits represent structured probability amplitudes that can simultaneously occupy multiple states. This confers a representational capacity and flexibility far beyond that of classical units, enabling QNNs to explore solution landscapes that grow exponentially with problem complexity.

Through this inherently parallel structure, QNNs simulate probabilistic reasoning, associative generalization, and context-dependent updating with an efficiency that resonates with biological cognition [31,32]. Importantly, these models do not assert that the brain is a quantum computer. Rather, they illustrate that quantum algebra provides a natural mathematical language for modelling cognitive phenomena such as rapid adaptation, contextual reframing, and flexible decision-making—phenomena that classical neural networks often struggle to reproduce with fidelity [8].

Aerts et al. [33] provide a concrete instantiation of this approach, adapting an abstracted quantum mechanical representation, with superposition, collapse, and entanglement, to model how concepts are actualized through interaction with context. They demonstrate that Bell inequalities are violated in the relationship between abstract concepts and their instances, and that quantum interference and superposition can account for well-documented cognitive abnormalities such as the conjunction fallacy and the disjunction effect, which classical probability frameworks fail to explain.

### 6.2. Quantum Tensor Networks: Hierarchies, Abstraction, and the Binding Problem

A parallel development stems from quantum tensor networks, mathematical structures devised initially to describe entangled many-body systems. Here, the emphasis is not on qubits per se but on representing complex interactions across hierarchical scales with remarkable computational efficiency [34]. Tensor networks can encode long-range dependencies, nonlinear relationships, and the integration of distributed features into unified percepts—core challenges that classical models tend to address only through ad hoc architectural modifications.

Tensor frameworks thus enable scalable representations of cognitive operations such as abstraction, recursion, and the binding of disparate sensory or conceptual elements into coherent wholes. In doing so, they blur the conceptual boundary between metaphor and mechanism. They do not imply that the brain literally implements tensor contractions, but they demonstrate that the mathematical structures underlying quantum physics possess an expressive power well suited to modelling cognition at levels of complexity where classical architectures saturate [1].

### 6.3. The Neurobiological Challenge: Connecting Formalism to Physiology

Despite their elegance, the connection between quantum-inspired computation and biological implementation remains tenuous. No empirical evidence currently shows that neurons or neural ensembles perform quantum logic operations or sustain entanglement in vivo. The difficulty arises from scale: quantum states typically evolve on femtosecond timescales and nanometer spatial resolutions—orders of magnitude finer than the temporal and spatial windows accessible to macroscopic neuroimaging tools such as EEG, MEG, or fMRI [17]. Bridging this explanatory gulf will require either the development of new neurotechnologies capable of resolving quantum signatures or deeper theoretical work articulating how quantum-like computation might emerge from classical neural systems through higher-order dynamics.

### 6.4. Emerging Empirical Signals

Although experimental evidence remains preliminary, several studies have begun testing predictions inspired by quantum-theoretic frameworks. EEG paradigms using quantum-randomized or entanglement-structured stimuli have demonstrated anomalous synchronization patterns and anticipatory neural responses, which are claimed to challenge classical learning models [20]. However, the use of the terms “quantum-randomized” or “entanglement-structured” must not be interpreted as suggesting that such an EEG study proposes that quantum entanglement occurs in the brain. Modified MRI protocols sensitive to zero-quantum coherence (ZQC) have reported state-dependent signal fluctuations that align with transitions in consciousness and have been interpreted as possible markers of state-dependent signal components, with some authors proposing (controversially) coherence-related interpretations [17]. These findings remain tentative, and replication will be crucial, but they indicate that quantum-informed hypotheses can now be subjected to empirical scrutiny using adapted neurotechnological tools.

### 6.5. Speculative Neurobiological Substrates

Beyond computational analogues, a growing set of neurobiological hypotheses proposes substrates capable of supporting non-classical interactions. Endogenous biophotonic processes within myelinated axons may produce entangled biphotons, facilitating long-range correlations that transcend synaptic connectivity [14,15]. More recent models treat the myelin sheath as a cylindrical optical resonator capable of sustaining coherent field modes and potentially enabling entanglement-mediated communication across neural structures [14]. These proposals are speculative, but they provide concrete predictions for future experiments, particularly those employing quantum-optical detectors and entanglement-sensitive imaging systems.

### 6.6. A Foundational Question: Mechanism or Metaphor?

The convergence of quantum-inspired computation and cognitive modelling raises a central epistemological question: Do these models succeed because the brain implements quantum mechanisms, or because quantum mathematics provides a structurally superior framework for describing systems characterized by uncertainty, contextuality, and global integration? At present, QNNs and tensor networks operate mainly as heuristic tools—powerful abstractions that emulate cognitive functions without making strong claims about neural implementation. Nevertheless, their unexpected fidelity in modelling complex behavior forces us to consider the possibility that cognition, whether grounded in quantum substrates or not, obeys principles that classical computation struggles to express.

Recent work in quantum psychology has advanced formal analogies between quantum principles and emotional or cognitive dynamics, proposing that superposition, entanglement, tunneling, and decoherence provide structurally rich frameworks for modeling ambiguity, emotional complexity, and contextual decision-making [35]. Importantly, these approaches do not claim that neural tissue implements physical quantum states. Rather, they employ Hilbert-space formalisms and quantum-probabilistic structures as mathematical and conceptual tools for describing psychological phenomena that resist classical linear models.

While such frameworks can generate computational simulations and yield novel representational architectures, their status remains formal rather than mechanistic. The use of quantum terminology in psychological modeling carries the risk of category errors, particularly if metaphorical mappings are conflated with biological claims. Moreover, the macroscopic scale of neural systems and the absence of empirical evidence for sustained coherence or entanglement in vivo require that these models be interpreted with epistemic restraint.

Thus, quantum-psychological approaches may offer explanatory value at the level of structure and representation, but they do not constitute evidence for quantum physics in the brain. Their contribution lies in expanding the mathematical vocabulary available to cognitive science, pending rigorous empirical validation.

A complementary line of work develops quantum-like cognition from rough-set theory and ambiguous representations, showing that paired Bayesian and inverse-Bayesian inference processes can generate logical structures corresponding to orthomodular lattices rather than classical Boolean algebras [36]. In this framework, ambiguity in representation—implemented through dual mappings constrained by inhibitory structure yields a non-classical logical space formally analogous to quantum logic. Importantly, these results are mathematical and structural, not physical: they demonstrate that certain cognitive architectures can be modeled using orthomodular lattices without implying that neural substrates instantiate Hilbert-space quantum states. The proposed “quantum-like” behavior thus arises from representational ambiguity and non-Boolean logic rather than from experimentally verified quantum coherence in the brain. While conceptually provocative, the biological implementation of such structures remains unestablished, and the interpretation should therefore be restricted to the level of formal cognitive modeling.

A related conceptual approach adapts Schrödinger’s cat into a modified “cat and mouse” thought experiment to illustrate the reciprocal relationship between Access consciousness (A-C) and Phenomenal consciousness (P-C) [37]. In this framework, the act of observation is treated analogously to measurement-induced collapse, so that direct interrogation of P-C renders it inaccessible, while A-C provides only retrospective, indirect signatures. The model introduces a “Consciousness Constant” to formalize the mutually exclusive dynamics of A-C and P-C and applies the analogy to clinical states such as delirium. Importantly, this work does not posit physical quantum processes in neural tissue; rather, it employs quantum thought experiments as heuristic and philosophical tools for clarifying measurement asymmetries in consciousness research. As such, its relevance is conceptual rather than mechanistic, and it should not be interpreted as evidence for quantum substrates of conscious experience.

A recent theoretical development by Wiest and Puniani [38] argues that temporally deep active inference, particularly the requirement to evaluate multiple future trajectories, may be mathematically equivalent to the quantum path integral formalism. The authors contend that realistic conductance-based spiking neuron models face substantial challenges in implementing rapid linear summation, model averaging, and path integration on sub-second timescales. They propose that quantum dynamics, owing to their intrinsic path-integral structure and linear superposition of probability amplitudes, could provide a natural physical implementation of the optimization required by active inference. In this framework, the combinatorial explosion inherent in classical sampling or attractor-based models would be resolved by the fundamental dynamics of quantum systems rather than by large-scale neural enumeration.

However, this proposal remains theoretical. The claimed equivalence between free energy minimization and the least action principle does not, by itself, establish that neural systems instantiate quantum path integrals. The critique of classical neural plausibility relies on heuristic estimates of neuron numbers, convergence times, and sampling speeds rather than definitive biophysical impossibility proofs. Moreover, demonstrating mathematical correspondence between inference algorithms and quantum formalism is distinct from demonstrating biological quantum coherence, entanglement, or collapse dynamics in neural tissue. Accordingly, the quantum active inference hypothesis should be interpreted as a mechanistic proposal motivated by computational considerations rather than as empirical evidence for the claim that consciousness depends on quantum-mechanical substrates.

In a companion article, Wiest and Puniani [39] extend this argument by proposing that Orchestrated Objective Reduction (Orch OR) in intraneuronal microtubules provides a mechanistic basis for the empirically observed discreteness of perceptual cycles. They review psychophysical evidence for non-overlapping temporal windows of conscious integration and argue that classical neural process models lack a biologically demonstrated mechanism for such discontinuous updates. The authors further cite studies reporting room-temperature microtubule resonances, anaesthesia-related modulation of microtubules, and MRI-based evidence of nonclassical brain signals, all of which support a quantum-microtubule substrate. However, these interpretations remain controversial. Direct in vivo demonstrations of long-lived entanglement gravitationally induced objective reduction (or collapse-based) neural computation have not yet been established. The proposed mapping between Orch OR dynamics and active inference, therefore, remains a speculative but formally articulated mechanistic hypothesis rather than a confirmed biological implementation.

Taken together, the intersections among quantum computing, tensor network theory, and neuroscience are reshaping the conceptual foundations of cognitive science. Quantum neural networks and tensor-based architectures provide models capable of capturing learning, memory, perception, and decision-making with an elegance and expressiveness that surpass classical approaches. Whether these frameworks reflect actual biological mechanisms or remain sophisticated metaphors is an open question—but one that is increasingly amenable to empirical investigation. As quantum technologies advance and neurobiology begins to integrate tools capable of probing coherence and entanglement at biologically relevant scales, the inquiry into whether quantum principles contribute to cognition may soon shift from philosophical debate to testable science.

Extending the biophotonic hypothesis, Schiffer [27] proposes a Four-Field Quantum Model in which coherent biophoton emissions serve as the mechanistic interface coupling structured brain information to four postulated fundamental quantum fields, Life, Subjective, Awareness, and Memory, via symmetry-breaking interactions. Clinical evidence from unilateral transcranial photobiomodulation (UtPBM), informed by Dual-Brain Psychology, is cited as preliminary support, though the proposed fields remain speculative and the model awaits direct experimental validation of the coherence effects it invokes.

## 7. Experimental Evidence and Challenges

Throughout this section, we treat “quantum-like” behavioral structure as conceptually distinct from claims of physical quantum states in neural tissue, and we reserve mechanistic language (coherence, entanglement, collapse) for contexts where an operational witness protocol and strong classical null models are available.

Empirical efforts to investigate whether quantum processes contribute to consciousness or cognition remain in their infancy (Table 2). Across neuroimaging, behavioral paradigms, and biophysical modeling, emerging findings are provocative yet preliminary. Accordingly, we distinguish carefully between results that motivate formal quantum modelling, results that suggest non-classical physical correlations, and mechanistic claims that would require direct evidence of biologically instantiated coherence or entanglement. Here, we synthesize the main experimental directions, highlighting both their conceptual promise and the substantial methodological challenges that must be addressed before firm conclusions can be drawn.

### 7.1. MRI-Based Indicators of Non-Classical Dynamics

**Figure 2:**
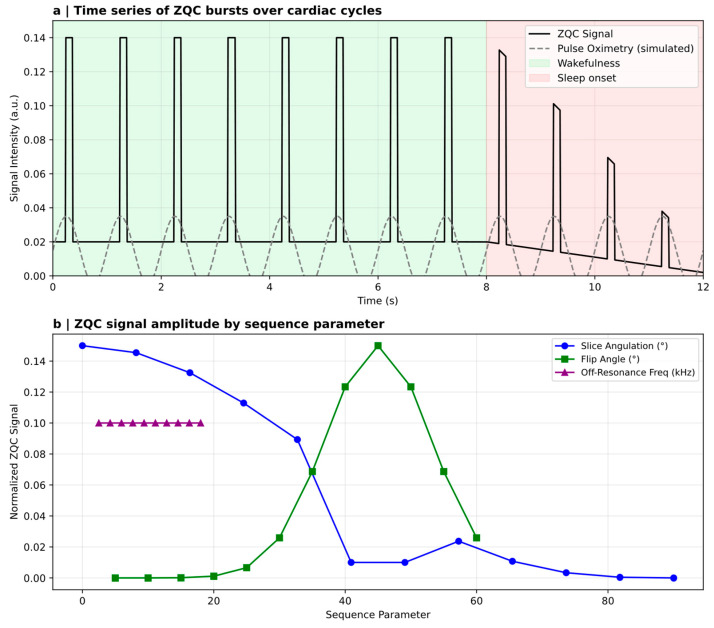
Time-resolved and sequence-dependent signatures of non-classical signal dynamics detected via zero-quantum-coherence (ZQC) MRI. (**a**) Time-resolved ZQC bursts. Echo-planar imaging, optimised to emphasise long-range zero-quantum coherence, reveals discrete, heartbeat-synchronised signal bursts during wakefulness. The time series illustrates high-amplitude, narrow ZQC peaks that align with cardiac cycles and diminish during the transition to sleep. Classical single-quantum coherence (SQC) contributions are suppressed by local magnetization saturation, leaving a residual component with a characteristic temporal profile (~180–450 ms). The attenuation of these bursts during reduced awareness suggests a functional dependence on conscious state rather than on vascular or motion artefacts. (**b**) Dependence on MRI sequence parameters. ZQC amplitude varies systematically with acquisition parameters. Signal intensity peaks at specific slice angulations—maximal outside the magic angle—consistent with dipole–dipole interactions rather than SQC effects. Flip-angle dependence reveals a maximum near 45°, distinct from the Ernst angle optimized for SQC, further indicating a coherence-based mechanism. Off-resonance manipulations show minimal attenuation, arguing against time-of-flight, slice-profile, or magnetization-transfer artefacts. Collectively, these parameter-dependent signatures point to a reproducible, non-classical signal component whose magnitude and state-dependence align with the ZQC framework. This figure is informed by the experimental paradigm introduced by Kerskens and López Pérez [17] but does not reproduce copyrighted material.

One of the most discussed candidates for quantum signatures in the brain is the observation of zero-quantum coherence (ZQC)-like signals in human MRI time series. Using pulse sequences designed to minimize classical intermolecular contributions, Kerskens and López Pérez reported heartbeat-locked signal bursts that lacked correlates in conventional single-quantum contrasts and varied with vigilance state and subjective reports of sleep onset in a subset of participants [17]. The authors interpreted these short-lived, awareness-dependent events as potential indicators of non-classical correlations, drawing inspiration from entanglement-witness logic while emphasizing the difficulty of excluding classical explanations in vivo (Figure 2).

This work offers a highly speculative attempt to operationalize a non-classicality-motivated MRI protocol that poses important technical challenges. Physiological confounds—such as motion, susceptibility fluctuations, and pulsatile hemodynamics—remain difficult to exclude fully. The absence of replication across independent scanners and laboratories further limits interpretability. Moreover, demonstrating that a non-classical signal constitutes entanglement rather than unusual but classical spin dynamics requires operational criteria more stringent than currently achievable. At present, the reported signals should be treated as intriguing anomalies rather than as demonstrations of entanglement or macroscopic quantum coherence in neural tissue. Nonetheless, the methodological innovation illustrates a pathway by which neuroimaging may contribute to evaluating quantum hypotheses.

### 7.2. Entanglement-Structured Learning and Neuroplasticity Markers

**Figure 3:**
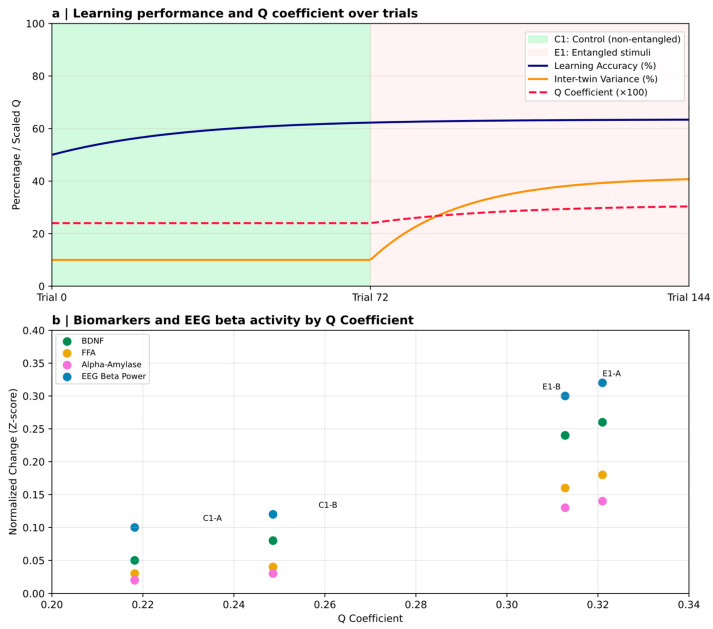
Behavioral, neurophysiological, and biomarker correlates of entanglement-structured learning in monozygotic twins. (**a**) Learning performance and variance structure. In a 144-trial implicit learning paradigm, twins completed tasks involving either non-entangled (C1) or entangled (E1) visual stimuli. Under entangled conditions, learning accuracy increased progressively relative to controls, producing a 13.5% improvement by the final trial block. Inter-twin response variance rose selectively in the entangled condition, reaching 31.6%, suggesting greater divergence in individual learning trajectories. The Quantum Multilinear Coefficient (Q)—used to index variance attributable to entanglement-specific task structure—was consistently higher in entangled trials (E1-A and E1-B) compared with non-entangled controls (C1-A and C1-B). Together, these behavioral measures indicate that entanglement-structured stimuli modulate both performance and variance within genetically identical pairs. (**b**) Biomarker and electrophysiological correlates. Peripheral and central physiological measures aligned with the behavioral effects of entangled stimuli. Increases were observed in biomarkers associated with neuroplasticity and arousal, including brain-derived neurotrophic factor (BDNF), free fatty acids (FFA), and salivary alpha-amylase. Electrophysiologically, entangled conditions evoked enhanced beta-band (13–30 Hz) power over frontal regions, consistent with increased cognitive integration and implicit learning. These biomarker and EEG changes scale with the Q-coefficient, indicating convergence between behavioral, physiological, and electrophysiological indices of entanglement-structured learning. This figure draws upon the experimental framework and findings reported by Escolà-Gascón [20] but does not reproduce copyrighted material.

A very different experimental strategy incorporates quantum structure directly into cognitive tasks. Escolà-Gascón [20] embedded entangled versus non-entangled qubit circuits as generators of stimulus contingency structure into visual stimulus contingencies within an implicit learning paradigm administered to monozygotic twins. Using the newly introduced Quantum-Multilinear Integrated Coefficient (Q), the study reported that entangled stimulus configurations accounted for 13.5% of the variance in accuracy and up to 31.6% of the variance in twin response divergence, with corresponding shifts in biomarkers of plasticity, including BDNF, free fatty acids, and α-amylase. These findings were interpreted as evidence that entanglement in the task’s stimulus-generation structure may modulate learning-related performance metrics (Figure 3). However, such effects, even if robust, would not by themselves establish entanglement or other quantum physical states in neural processing. Hence, such interpretations warrant caution.

A conservative interpretation is therefore that the study by Escolà-Gascón [20] probes the sensitivity of learning to non-classically generated task statistics, rather than the presence of non-classical brain states. Claims about “quantum entanglement in consciousness” should be considered out of scope unless a biological entanglement witness is defined and satisfied.

The Q-coefficient captures variance aligned with a mathematically non-classical structure, but it does not provide direct evidence of quantum physical processes in neural tissue. Twin-based paradigms introduce complex dependence structures that require rigorous controls for expectancy and shared-environment effects. Perhaps most critically, entanglement in the stimulus design does not imply entanglement in neural processing. These experiments are notable for their conceptual originality—they treat entanglement not as a hypothetical brain property but as an experimentally manipulable variable. Nevertheless, the leap from quantum-defined “task relations” to quantum-enabled “neural mechanisms” remains unsubstantiated.

### 7.3. Quantum-like Implicit Learning and Anomalous Information Anticipation

A related body of work examines whether certain forms of learning and prediction exceed the explanatory scope of classical conditioning. In a study of “anomalous information anticipation” (AIA), Escolà-Gascón [19] combined continuous flash suppression, quantum random event generators, and high-density EEG to examine whether participants could predict outcomes of sensory stimuli that were intended to be perceptually inaccessible, conditional on the effectiveness of masking and the exclusion of subtle sensory leakage. Behavioral accuracies exceeded chance, with explained variances ranging from 25% to 48%, and neurophysiological markers in occipital, parietal, and medial temporal regions corresponded to implicit learning circuits.

These results may challenge canonical assumptions about the necessity of spatiotemporal contingency for associative learning. Nevertheless, alternative interpretations persist. Subtle sensory leakage, implicit biases, and contextual expectation effects are notoriously difficult to eliminate in implicit anticipation/presentiment-style paradigms. Moreover, although the data were modelled using quantum-like mathematical frameworks, such formalisms do not, in themselves, entail quantum physical substrates. The findings highlight that certain cognitive phenomena—especially those involving uncertainty, contextuality, or non-Markovian structure—may in some cases be more parsimoniously captured by quantum probability models than by classical ones. Whether this reflects the brain’s underlying physics or simply its computational architecture remains unresolved.

Bell-type inequalities, originally formulated in physics to test whether local realistic (classical) models can explain observed correlations, provide operational criteria for distinguishing classical probability structures from non-classical ones. In their temporal form—known as the Leggett–Garg or Temporal Bell inequalities—they test the assumption of macrorealism, namely that a system possesses definite properties at all times independent of measurement. Violations of these inequalities indicate that observed correlations cannot be fully accounted for by classical stochastic models satisfying non-invasive measurability and temporal consistency.

Applying this framework to cognition, Waddup et al. [41] tested a Temporal Bell inequality in a memory paradigm using change judgments across sequential time points. In one experiment, they observed a pattern of correlations consistent with a violation of the Leggett–Garg inequality, which they interpreted as evidence against macrorealism in reconstructive memory processes. Importantly, such violations indicate quantum-like probabilistic structure at the level of behavioral correlations; they do not demonstrate physical entanglement, neural superposition, or quantum coherence in brain tissue. Moreover, the interpretation depends on assumptions such as non-invasive measurability and homogeneous participant preparation, both of which are methodologically challenging in psychological contexts. Accordingly, Temporal Bell violations in cognition should be understood as constraints on classical probabilistic models rather than as evidence for quantum neurobiology.

### 7.4. Microtubule Coherence and Orchestrated Objective Reduction

At the cellular scale, the Orch OR theory posits that quantum-coherent states form within neuronal microtubules and undergo objective reduction, thereby generating discrete conscious moments [42]. The theory integrates quantum gravity proposals with cytoskeletal dynamics, suggesting that tubulin conformational states function as quantum-computing elements whose collective collapse contributes to the unity and indivisibility of conscious experience.

Although Orch OR has inspired significant interdisciplinary discussion, empirical support for long-lived microtubule coherence at physiological temperatures remains lacking. Decoherence estimates vary by orders of magnitude, and experimental evidence for microtubule-based quantum processing has not been demonstrated in vivo. Recent proposals—for example, using isotope-dependent anaesthetic effects [16] to probe quantum-sensitive biological mechanisms—offer a testable framework but remain at the conceptual stage. Overall, Orch OR provides a rich theoretical lens but awaits decisive biological validation.

### 7.5. Contextuality, Interference, and Classical Explanations of Quantum-like Cognition

Several researchers caution that many reported “quantum” effects in psychology and neuroscience may arise from contextuality rather than physical quantum processes. Classical systems capable of interference, phase relationships, or oscillatory interactions can reproduce patterns that resemble quantum probability structures. As Acacio de Barros and Suppes [7] argue, contextuality—rather than entanglement or superposition—may be the true driver of quantum-like cognitive effects, including apparent violations of classical probability axioms in behavioral modelling, where such claims depend on the specific experimental design and the appropriateness of the classical null model.

This perspective suggests that quantum formalisms may succeed as descriptive or predictive tools without implying quantum substrates. It also offers a potentially parsimonious explanation for behavioral effects observed in AIA studies or decision-making paradigms: cognitive systems that integrate information across incompatible contexts may naturally exhibit interference-like structure. Thus, while quantum models can be powerful, their empirical success does not, in itself, validate quantum biological mechanisms.

Nevertheless, Aerts et al. [33] argue that the contextual nature of conscious experience aligns structurally with quantum mechanics. Their demonstration that Bell inequalities are violated in concept–instance relationships suggests that quantum formalism captures genuine structural features of cognition, not merely surface-level interference patterns, even when no physical quantum substrate is implied.

### 7.6. Stochastic Reduction Models and the Physics of Collapse

Another relevant theoretical development concerns stochastic extensions of the Schrödinger equation. Brody and Hughston [40] describe how nonlinear stochastic dynamics can induce spontaneous state reduction, offering a continuous, physically motivated alternative to the instantaneous collapse postulate. Although these models are not specific to biology, they provide mathematical tools for thinking about how quantum-like collapse dynamics might unfold in noisy, open systems, without implying that such dynamics occur in neural tissue.

To date, no evidence demonstrates that neurons or molecular substrates operate according to such stochastic reduction mechanisms. Nonetheless, these frameworks may inform future modelling of neural systems if quantum coherence were ever empirically established at biologically meaningful scales.

### 7.7. Cross-Cutting Challenges in Evaluating Quantum Hypotheses

Across these diverse research programs, several broad challenges emerge. First, replication remains limited. Many findings—from ZQC signals to entanglement-structured learning—have not yet been reproduced under blinded, preregistered, or multicenter conditions. Second, distinguishing quantum physical processes from quantum-like models is essential. The brain may implement computations that “mathematically resemble” quantum processes without hosting physical quantum states. Third, biological plausibility requires demonstration of neural structures capable of maintaining coherence or entanglement. Proposed candidates such as microtubules, nuclear spins, or photonic axonal channels remain hypothetical. Fourth, measurement limitations constrain progress. Quantum events occur at temporal and spatial scales far beyond the resolution of current neuroimaging, electrophysiology, or optical probes. Finally, conceptual clarity is needed. Different theories invoke quantum mechanics for mechanistic, computational, or phenomenological reasons, and these must not be conflated.

Taken together, existing evidence does not demonstrate that consciousness requires quantum processes, but it does justify ongoing investigation. MRI studies suggest the possibility—still controversial—of state-dependent signal components that some authors interpret as potentially non-classical. Behavioral and EEG data have been reported to show patterns that certain forms of learning and anticipation align more naturally with quantum probability structures than with classical models. This supports the utility of quantum formalisms as descriptive tools, but it does not establish a quantum physical substrate. Microtubule-based frameworks offer ambitious, testable hypotheses linking quantum physics to conscious experience, though direct biological evidence remains absent.

Furthermore, theoretical work on contextuality and stochastic dynamics provides interpretive tools for understanding both quantum-like cognition and potential future quantum-biological discoveries. The field is therefore best understood as one in transition: moving from largely speculative theorization toward increasingly empirical, mechanistically explicit research programs. Advances in quantum-sensitive imaging, isotope-specific pharmacology, nanoscale magnetometry, and high-precision behavioural paradigms may soon enable tests previously inaccessible, including experiments explicitly designed to discriminate non-classical correlations from high-dimensional classical dynamics under preregistered, adversarial conditions. The challenge—and opportunity—lies in designing experiments capable of distinguishing genuinely non-classical physical correlations from the many classical processes that can mimic their signatures. We employ the words “non-classical physical correlations” instead of “quantum physics in the brain”, given that it is crucial to be cautious when considering quantum physics in the context of the brain’s physiological processes.

## 8. Discussion

The proposition that quantum processes may contribute to consciousness has transitioned from speculative conjecture to an increasingly structured, though still highly debated, domain of scientific inquiry. This transition has been shaped not by sensational claims but by the gradual accumulation of theoretical, computational, and empirical signals suggesting that classical neuroscience faces ongoing challenges in explaining certain structural features of conscious experience. Conscious experience—unitary, coherent, contextually embedded, and globally integrated—exhibits structural features that classical models continue to investigate with increasing sophistication. Quantum theory, with its principles of non-separability, contextuality, and global constraint, offers a vocabulary that resonates more naturally with these properties and may therefore help illuminate aspects of consciousness that remain opaque within classical frameworks. Importantly, resonance at the level of formal structure does not imply mechanistic equivalence at the level of physical substrate.

Zero-quantum-coherence (ZQC) MRI has provided one of the most provocative empirical openings. By suppressing conventional nuclear magnetic resonance components, this protocol reveals heartbeat-locked signal bursts that have been reported to vary with vigilance state in limited samples. If these fluctuations reflect coherence-sensitive interactions rather than vascular or diffusion artefacts, they may constitute candidate state-dependent signal components requiring explanation beyond standard single-quantum models. However, extraordinary claims require extraordinary verification. Rigorous replication across scanners, laboratories, and physiological manipulations is essential before ZQC can be interpreted as evidence for non-classical neural dynamics. The field must resist the temptation to overinterpret; the stakes of being wrong are high, but so too are the risks of prematurely attributing complex classical dynamics to quantum origins.

Behavioral and electrophysiological findings from entanglement-structured learning paradigms raise equally compelling questions. The observation that quantum-relational stimulus structures can modulate behavioral accuracy, inter-individual variance, and indices of neuroplasticity suggests that the brain may be sensitive to patterns of information that extend beyond classical statistical correlations. However, sensitivity to non-classically generated task structure does not demonstrate the presence of non-classical neural states. The Quantum Multilinear Coefficient (Q) represents an innovative attempt to quantify such structure-aligned variance in cortical signals, though its interpretation remains provisional. These findings do not yet demonstrate quantum processes in neural tissue. However, they motivate further examination of whether classical learning models are sufficient under all contextual conditions and highlight the need for models that can accommodate non-local, contextual forms of information integration.

Classical neuroscience has excelled at mapping localized mechanisms—such as synaptic transmission, oscillatory coordination, and large-scale network dynamics. However, consciousness is not merely the sum of these components. It presents as a coherent whole, in which perception, memory, emotion, and intention are bound together into a unified experiential field. Whether local mechanisms are sufficient to account for this global unity remains an open question under active investigation. Quantum-inspired models, whether invoking superposition-like representational richness, entanglement-like correlations, or collapse-like transitions, explicitly address the structural features of consciousness that classical approaches struggle to explain.

In this regard, Abramov et al. [25] showed that global neural complexity assessed via q-statistics exceeded the sum of local complexities, consistent with non-additive, irreducible brain organization. Whether such non-additivity reflects quantum-physical non-separability or classical nonlinear dynamics remains an open question.

The causal potency of consciousness presents a central challenge for evolutionary accounts. Quantum indeterminism naturally accommodates such potency, whereas deterministic classical dynamics would render emergent consciousness causally ineffective. Whether quantum-level agency can be demonstrated in biological neural systems remains to be established [26].

A complementary perspective proposes that biophoton coherence mediates the coupling between neural activity and postulated quantum fields responsible for subjective experience, with hemisphere-specific neuromodulation (UtPBM) providing preliminary clinical evidence for state-dependent shifts in consciousness. Although this four-field framework lacks direct empirical validation at the quantum level, it illustrates the growing interest in testable, field-based models of consciousness [27].

An extension of this reasoning draws on Turing mathematics to argue that biological information generation requires agency that transcends computation, with quantum measurement by cognitive cells proposed as the mechanism through which organisms produce order from disorder. If substantiated, this would position consciousness not as an emergent epiphenomenon but as integral to the physical fabric of biological reality, though empirical validation of cells acting as quantum observers remains an open challenge [28].

The formation of a superposition may also coincide with a moment of agency; the homeostatic correlation between pleasant feelings and survival conducive behaviors is difficult to explain if organisms are deterministic automata. A proposed experimental program to replicate xenon isotope anesthetic effects on brain organoids and Drosophila with high statistical power offers a near-term, empirically tractable test of whether nuclear spins contribute to the physical substrate of consciousness. However, results remain forthcoming [16].

A recent conceptual proposal by Vitas [43] advances a definitional reformulation of consciousness as “an emergent property of a far-from-equilibrium system of quantum particles sustained by an autopoietic system and capable of processing, transforming, and accumulating information acquired from the environment.” This framework derives consciousness from a generalized definition of life and emphasizes thermodynamic non-equilibrium, information processing, and a quantum-level ontology. While philosophically ambitious and internally coherent, this proposal remains primarily conceptual rather than empirical. It does not provide operational criteria for detecting quantum coherence, entanglement, or other non-classical correlations in neural tissue, nor does it establish how such quantum-particle descriptions would be distinguished experimentally from classical chemical and electrochemical processes. The move from “chemical system” to “system of quantum particles” represents an ontological rephrasing rather than a demonstrated mechanistic necessity. As such, the proposal should be interpreted as a speculative definitional hypothesis that invites formal clarification and empirical specification, rather than as evidence that consciousness depends on quantum-mechanical substrates.

Experimentally discriminable predictions must ultimately accompany any definition that embeds quantum particles at the ontological level; otherwise, the appeal to quantum theory risks becoming a universal reduction that applies equally to all matter, living or non-living.

Opponents of quantum models often invoke decoherence as a definitive objection, arguing that the brain’s warm, noisy environment is inhospitable to sustained quantum processes. Research in quantum biology has demonstrated that certain quantum effects can operate under physiological conditions in specific molecular systems. Long-lived coherence in photosynthetic complexes and entangled radical pairs in avian magnetoreception demonstrate that biological systems can, under appropriate structural and dynamical conditions, exploit quantum effects at physiological temperatures. These cases do not prove that the brain maintains coherence or entanglement, but they remove a key conceptual barrier: the presumption of impossibility. They establish plausibility in principle, not evidence in the neural domain.

The technological challenge, rather, lies in measurement. Quantum processes unfold at spatiotemporal scales far below the resolution of traditional neuroimaging and electrophysiology. The emerging suite of quantum-enhanced tools—NV-diamond magnetometers, entanglement-sensitive spectroscopy, hybrid optical-neural interfaces, and next-generation ZQC protocols—promises, for the first time, to probe whether neural systems generate, propagate, or respond to quantum-level correlations.

The integration of these technologies with optogenetics, organoids, and computational modelling may soon enable causal testing of hypotheses previously methodologically unreachable. Whether these tools will reveal genuine non-classical dynamics or instead refine classical models remains an empirical question. The theoretical landscape is evolving in parallel. Models of microtubular coherence, myelin-based photonic waveguides, nuclear-spin networks, and contextual quantum probability offer diverse routes for embedding non-classical dynamics into neural function. Quantum neural networks and tensor architectures demonstrate that quantum mathematics provides expressive models of learning, abstraction, and decision-making, regardless of whether the brain physically implements such dynamics. This duality—quantum models as both mechanistic hypotheses and computational metaphors—illustrates the field’s current epistemic state: conceptual expansion coupled with empirical uncertainty. At present, the balance of evidence supports cautious exploration rather than mechanistic commitment.

Recent developments have introduced additional physics-inspired frameworks that further expand the landscape of quantum-related approaches without requiring genuine quantum coherence. In particular, lattice field theory, originally developed in quantum field theory and particle physics, has been adapted to model spatiotemporal neural dynamics by discretizing neural activity on a spacetime lattice [44]. Within this framework, neuronal states—such as action potentials in biological networks or node activations in artificial neural systems—are encoded as binary variables on lattice sites, enabling their formal treatment using tools analogous to those employed in quantum many-body systems. This representation allows the construction of effective theories through coarse-graining procedures, in which macroscopic neural dynamics emerge from underlying discrete interactions, conceptually paralleling renormalisation approaches in statistical and quantum field theory [44].

Importantly, these models provide a unifying computational framework that bridges statistical physics, neural network theory, and quantum-inspired formalisms. By leveraging lattice-based formulations, they enable the analysis of high-dimensional, strongly interacting neural systems in parameter regimes that are often intractable for conventional approaches, including both biological spiking networks and deep learning architectures. Moreover, Bardella et al. highlight that such frameworks may facilitate cross-fertilization between disciplines, including the application of quantum computing techniques to neural modeling and, conversely, the use of neural systems as substrates for simulating complex physical theories [44].

Despite these promising features, it is important to emphasize that lattice field theory-based approaches remain primarily theoretical and computational. Their current contribution lies in providing a principled formalism for describing emergent spatiotemporal structure in neural activity, rather than in demonstrating biologically instantiated quantum processes. As such, they are best understood as part of a broader class of quantum-inspired methodologies that extend the analytical toolkit of neuroscience, while remaining compatible with classical neurophysiological mechanisms.

Within this broader context, an important conceptual distinction arises between models that posit genuine quantum physical processes in the brain and those that employ quantum formalism as a descriptive framework. Quantum brain hypotheses propose that phenomena such as coherence, entanglement, or wave-function collapse are physically instantiated in neural substrates and play a causal role in conscious processing. In contrast, quantum-like approaches adopt the mathematical structure of quantum theory—particularly Hilbert space representations and quantum probability—to model cognitive phenomena without assuming that the brain operates as a quantum physical system [45].

From a cognitive science perspective, this distinction aligns with the notion of levels of explanation. As emphasised in the quantum cognition literature, including recent syntheses, probabilistic models—whether classical or quantum—primarily operate at the computational level, aiming to characterise the principles governing behaviour rather than the underlying biological implementation [46]. In this framework, quantum probability theory is treated as a generalisation of classical probability theory that better captures how agents assign probabilities under uncertainty, particularly in situations involving contextuality, ambiguity, and sequential effects [46]. Thus, quantum-like models should be understood as formal descriptions of cognitive processes at an abstract level, not as direct claims about neural microphysics.

The rationale for this framework arises from robust empirical observations in cognitive psychology and decision science showing systematic violations of classical probability theory. These include order effects, conjunction and disjunction fallacies, and other context-dependent phenomena that cannot be fully captured by classical probabilistic models but are naturally accommodated within quantum probability through interference and contextuality [45]. In this sense, quantum-like models provide a principled formalism for representing non-classical statistical structure in cognition, offering explanatory coherence across diverse behavioral domains.

Importantly, this approach is explicitly operational. As emphasised by Khrennikov and others, the goal is not to explain the neurobiological origins of these statistical patterns, but to model them at the level of observable behaviour and information processing [45]. Correspondingly, concepts such as superposition, incompatibility, or entanglement are interpreted as epiphenomenal descriptors of behavior, rather than physically instantiated properties of neural systems [46]. Mental states are thus represented as superpositions that encode uncertainty, and cognitive variables may behave as incompatible observables, giving rise to order-dependent effects and violations of the classical formula for total probability.

More recent developments have attempted to provide a tentative bridge between this formal framework and neurobiology. Models based on a quantum information representation of neuronal states suggest that the intrinsic uncertainty in electrochemical processes—such as stochastic ion-channel dynamics and variability in action potential generation—can be mapped onto superposition-like informational states [47]. Within this view, neuronal assemblies can be treated as open systems whose interaction with their environment leads to decoherence-like dynamics, with decision outcomes corresponding to stable states of this process [47]. However, even in these formulations, the quantum structure remains at the level of information representation and does not require sustained physical quantum coherence in neural tissue.

These perspectives indicate that quantum brain and quantum-like approaches operate at distinct explanatory levels. The former seek to identify physical quantum mechanisms underlying consciousness at the level of biological implementation, whereas the latter provide a mathematically consistent framework for describing cognitive phenomena at computational and algorithmic levels [46]. In this sense, they are not strictly competing paradigms but rather complementary approaches that address different aspects of the same problem.

At present, empirical support is considerably stronger for the quantum-like framework, which has demonstrated explanatory value across a range of behavioral phenomena. In contrast, quantum brain hypotheses remain largely theoretical and face substantial challenges related to decoherence, scalability, and experimental validation. Moreover, from the standpoint of cognitive theory, many of the features captured by quantum models—such as contextuality or interference—may ultimately reflect emergent properties of large-scale neural systems rather than signatures of underlying quantum physical processes [46]. Clarifying this distinction is essential to avoid conceptual conflation between formal modelling success and mechanistic explanation.

### 8.1. Limitations and Alternative Explanations

A strong classical counterposition was articulated by Baars and Edelman [48], who argued that while all biological processes are reducible to quantum events in principle, quantum mechanics does not necessarily provide the appropriate level of explanation for consciousness. They emphasize that conscious states are reliably differentiated from unconscious states at the level of large-scale cortico-thalamic dynamics, oscillatory regimes, and global integration, rather than at the level of microtubular or molecular events. Notably, they point out that microtubules and quantum interactions are ubiquitous across cells, including plant cells and brain regions not associated with conscious contents, thus failing to explain the empirical contrast between conscious and unconscious neural processes. From this perspective, the burden of proof remains with quantum-level accounts to demonstrate a distinctive, causally relevant contribution to conscious cognition.

The present body of research remains constrained by substantial methodological, theoretical, and interpretive limitations. First, replication across independent laboratories remains limited for many of the most provocative findings, including ZQC-based MRI signals and entanglement-structured learning paradigms. Without multi-site validation under blinded and preregistered protocols, apparent anomalies may reflect analytic flexibility, physiological confounds, or unmodelled classical dynamics.

Second, distinguishing quantum physical processes from quantum-like mathematical descriptions is essential. Behavioral violations of classical probability axioms, contextuality effects, or interference-like patterns can arise in entirely classical systems with complex nonlinear dynamics. The success of quantum probability models does not entail that the underlying neural substrate is quantum mechanical.

Third, biological plausibility remains an open challenge. Proposed substrates, including microtubules, nuclear spins, and photonic waveguides, lack direct in vivo demonstration of long-lived coherence, entanglement, or collapse dynamics at scales relevant to neural computation. The absence of operational entanglement witnesses in biological tissue significantly limits the validity of mechanistic claims.

Fourth, measurement resolution imposes severe constraints. Quantum phenomena typically manifest at femtosecond timescales and nanometer spatial resolutions, whereas most neuroimaging techniques operate at scales many orders of magnitude higher. Bridging this gap requires either radically improved instrumentation or theoretical accounts of how microscopic non-classical effects could scale to macroscopic neural signatures.

Finally, complex classical systems can mimic features often labelled “quantum,” including contextuality, nonlinearity, long-range correlations, and multifractality. Rigorous null modelling, adversarial collaboration, and formal separability criteria will be required to prevent misclassification of classical complexity as quantum structure.

A strong limitation in this field is the great number of speculations derived from models. A recent hypothesis extends collapse-based interpretations of quantum mechanics to propose that wavefunction reduction within specific “prime neurons” mediates free will through the action of a hypothetical “soul/spirit particle” [49]. In this framework, intentionality biases collapse probabilities, thereby altering ion-channel states and neural firing patterns. The proposal includes a formal mathematical operator for will-driven collapse and attributes exotic physical properties to the postulated particle, including ultra-weak coupling and resistance to decoherence. However, this account rests on multiple speculative premises, including the existence of a new particle not supported by current particle physics, the capacity to modulate collapse probabilities intentionally, and sustained quantum superposition in neuronal ion channels under physiological conditions. At present, there is no empirical evidence for such a particle, for intentional collapse bias, or for biologically implemented non-linear collapse dynamics. While the model is internally structured and mathematically articulated, it should be regarded as a metaphysical extension rather than a testable physical theory unless and until experimentally falsifiable predictions are operationalized and independently replicated.

A conceptual review by Wiest [50] argues that microtubules constitute a quantum substrate of consciousness and that Orch OR provides a unified solution to the binding problem and the epiphenomenalism problem. The article synthesizes anesthetic pharmacology, reports room-temperature microtubule resonances, and MRI-based findings interpreted as macroscopic entanglement, presenting these as convergent evidence for a quantum microtubule model. However, the paper introduces no new experimental data, and its interpretations remain debated. Direct in vivo demonstrations of long-lived entanglement, objective reduction, or causally efficacious quantum collapse in neural tissue have not yet been independently replicated under stringent controls. Accordingly, while the proposal is philosophically and theoretically ambitious, it should be regarded as a speculative integrative hypothesis rather than as an established empirical confirmation of a quantum substrate of consciousness.

For these reasons, current evidence should be interpreted as hypothesis-generating rather than hypothesis-confirming.

### 8.2. Challenges and Opportunities

The challenge, and opportunity, lies in designing experiments capable of distinguishing genuinely non-classical physical correlations from the many classical processes that can mimic their signatures. At the end of Section 7, we intentionally employed the term “non-classical physical correlations” rather than “quantum physics in the brain.” The latter phrase implies that neural tissue has already been shown to instantiate well-characterised quantum states, such as entanglement, long-lived coherence, or controlled-collapse dynamics, claims that would require operational criteria comparable to those used in controlled quantum-optical or condensed-matter systems. In contrast, “non-classical physical correlations” denotes a more modest and methodologically appropriate target: correlations that cannot be fully accounted for by separable classical models, stochastic nonlinear dynamics, or high-dimensional deterministic chaos, and that would satisfy clearly defined physical non-separability or contextuality criteria.

This distinction is particularly important because neuroscience currently operates under severe experimental constraints relative to laboratory quantum physics. In engineered non-living systems, quantum states can be isolated, cooled, shielded from environmental decoherence, and subjected to precise Bell-type inequalities, entanglement witnesses, or interferometric phase measurements. Living neural systems, by contrast, are thermodynamically open, metabolically active, and embedded within complex vascular, electromagnetic, and biochemical noise sources. As a result, the standards of evidence required to demonstrate entanglement or other strictly quantum phenomena in vivo must be carefully calibrated, without diluting their rigor.

Framing the problem in terms of non-classical physical correlations allows the field to advance incrementally and responsibly. It shifts the burden of proof from asserting “quantum brain” mechanisms to identifying measurable departures from well-specified classical null models. Only once such departures are robustly established and shown to satisfy operational non-classicality criteria would it be appropriate to escalate interpretive claims toward explicit quantum-mechanical mechanisms. Until then, methodological humility remains essential.

In this sense, the investigation of quantum-informed neuroscience should proceed not by ontological assertion but by progressively narrowing the space of viable classical explanations.

### 8.3. Future Directions and Interrogations

What emerges is a field defined not by consensus, but by courage—the courage to interrogate assumptions, to refine bold hypotheses with rigorous data, and to push neuroscience toward foundational questions it has historically avoided. Does consciousness require quantum mechanisms, or merely quantum mathematics? Are the global, unified properties of experience reflections of non-classical correlations, or can classical dynamics approximate these patterns under conditions of extreme complexity? Can coherence-like signals in MRI or quantum-structured learning effects in EEG be reconciled with classical nonlinear systems, or do they point toward deeper physical processes in the brain?

These questions do not diminish classical neuroscience; they extend it. They challenge the field to consider explanations that bridge the microscopic and the macroscopic, physics and phenomenology, mechanism and experience. Whether quantum principles serve as metaphors, mathematical tools, or mechanistic realities remains unresolved. Nevertheless, for the first time, the tools, theories, and empirical footholds exist to transform these questions into testable science.

If the coming decade reveals that quantum processes underlie consciousness, neuroscience will undergo a conceptual shift on par with the revolutions of relativity and quantum mechanics. If, instead, classical systems prove sufficient, the field will have refined its models and deepened its understanding of complexity, integration, and computation. Both outcomes are victories. What matters now is that we ask the right questions—and pursue them with the methodological precision, interdisciplinary collaboration, and intellectual ambition that a problem as profound as consciousness demands.

Recent theoretical work has proposed that conscious processing may arise at classical-quantum interfaces within neural tissue, particularly in calcium-dependent synaptic mechanisms [51], astrocytic hydro-ionic waves, and ion–water interactions in the extracellular milieu [52]. These models suggest that biologically regulated ionic dynamics, especially involving Ca^2+^, hydrogen ions, and structured interfacial water, could, under certain conditions, support transient coherence-like states that influence large-scale neural integration.

Importantly, such proposals do not demonstrate sustained quantum computation in the brain, nor do they establish entanglement or recoherence at macroscopic neural scales. Rather, they identify molecular and mesoscopic processes where non-classical correlations might be theoretically plausible. At present, these mechanisms remain hypothetical and require rigorous empirical discrimination from complex but fully classical electrochemical dynamics.

Future research should therefore prioritize:

- Quantitative modeling of calcium-mediated signal integration under realistic thermal conditions;
- Direct measurement of coherence lifetimes in neural microenvironments;
- Experimental tests capable of distinguishing structured ionic energy transfer from diffusion-based classical propagation;
- Adversarial null-model comparisons between nonlinear classical field dynamics and proposed non-classical effects.

Particularly promising would be experiments designed to evaluate whether structured proton conduction (e.g., Grotthuss-like mechanisms) or collective ionic phase transitions produce measurable signatures beyond classical stochastic biophysics. However, any claim of recoherence or entanglement in vivo must satisfy stringent operational criteria, including reproducibility across preparations and resistance to classical reinterpretation.

Thus, the path forward is neither dismissal nor affirmation but disciplined refinement. The concept of classical–quantum interfaces offers a heuristic framework for exploring multiscale integration in neural systems, yet its explanatory value will depend entirely on experimentally testable predictions and methodological rigor.

## 9. Conclusions

The question of whether quantum processes contribute to consciousness remains open. Current empirical findings do not establish that the brain sustains entanglement, long-lived coherence, or collapse dynamics relevant to neural computation. However, they do justify continued investigation into whether non-classical mathematical frameworks better describe certain neural or cognitive phenomena, and whether future experimental advances might reveal genuinely non-classical biological correlations.

The field stands at a methodological crossroads. One path leads toward premature ontological commitment, the other toward disciplined, testable exploration. The responsible trajectory lies in maintaining conceptual openness while enforcing strict evidential standards. Claims must scale with data. Mechanistic assertions must follow operational criteria. Quantum-like descriptions in neuroscience must not be conflated with quantum physical states as in non-living systems.

Whether the coming decade reveals that consciousness depends on quantum mechanisms or that carefully formulated classical models prove adequate, the investigation itself will deepen our understanding of integration, context, and complexity in neural systems. The aim is not to vindicate a quantum hypothesis but to determine, through rigorous experimentation and conceptual clarity, which explanatory framework most coherently accounts for conscious experience.

In that spirit, quantum neuroscience should be understood not as a declaration but as a disciplined inquiry.

## Figures and Tables

**Table 1:** Overview of quantum-related hypotheses of consciousness and representative experimental indicators.

Domain	Proposed Quantum Feature	Candidate Neural Mechanism	Representative Indicators	Key Challenges
Non-classical MRI dynamics	Non-classical spin correlations → ZQC-like coherence	Mesoscopic proton–spin networks	Heartbeat-linked ZQC bursts; awareness-dependent signal modulation	Artefact exclusion; entanglement not directly tested; replication needed
Entanglement-structured cognition	Entangled relational structure → variance amplification → enhanced learning	Network-level sensitivity to non-local task structure	Increased Q-coefficient; boosted learning variance; biomarker shifts	Task entanglement ≠, neural entanglement, expectancy and twin-based confounds
Quantum-like implicit learning (AIA)	Non-local prediction structure → implicit anticipation	Distributed implicit-learning circuits (occipital–parietal–MTL)	Above-chance AIA performance; trial-dependent learning acceleration; EEG correlates	Alternative classical models; need for blinded controls; no physical entanglement measurement
Microtubule quantum coherence (Orch OR)	Tubulin coherence → objective reduction → discrete conscious moments	Cytoskeletal microtubule lattices	Theoretical coherence thresholds; anesthetic isotope sensitivity predictions	Decoherence at physiological temperature; lack of in situ microtubule coherence evidence
Contextuality-based explanations	Contextuality → interference-like cognitive states	Classical oscillatory or field-based interactions	Violations of classical probability; interference patterns in decisions	Not uniquely quantum; classical contextuality is sufficient
Stochastic reduction frameworks	Noise-driven collapse → measurement-like behavior	Hypothetical micro-scale collapse processes	Energy-based stochastic collapse models	No biological demonstration; unclear neural relevance

Take-home message: Across neuroimaging, behavioral, and theoretical domains, converging evidence points to quantum-like or non-classical signatures in neural activity and cognition. Although not definitive proof of quantum physical processes in the brain, these findings provide testable frameworks that extend beyond classical neuroscience. Together, they motivate rigorous experimental programs aimed at determining whether non-classical dynamics—ranging from coherence and contextuality to entanglement-structured information processing—play a meaningful role in perception, learning, and consciousness. EEG, electroencephalography; ZQC, zero-quantum coherence; AIA, anomalous information anticipation; Q-coefficient, statistical index capturing variance aligned with entanglement-structured task relations; SSE, stochastic Schrödinger equation; Orch OR, orchestrated objective reduction; MTL, medial temporal lobe.

**Table 2:** Experimental evidence and challenges in quantum neuroscience.

Study/Model	Method	Key Findings	Interpretation	Limitations/Challenges
ZQC–MRI [17]	3T MRI → ZQC-sensitive sequences	Heartbeat-locked cortical signals → disappear in sleep/anesthesia	Awareness-dependent coherence-like activity	Vascular/diffusion artefacts possible → replication required
Twin EEG with entangled stimuli [20]	EEG → entangled vs. non-entangled stimuli → biomarker assays	↑ Accuracy, ↑ twin variance, ↑ plasticity biomarkers	Entangled task structure → behavioral & neuroplastic modulation	Q-coefficient novel → expectancy & twin confounds → reproducibility pending
Anomalous information anticipation (AIA) [19]	Continuous flash suppression → 3D EEG → 144-trial protocol	Predictive accuracy 25–48% → posterior activation	Quantum-like implicit anticipation → non-classical learning profile	No entanglement measure → small sample → need for blinded designs
Quantum interference models [7]	Quantum probability → cognitive modelling	Captures contextual decisions → classical rule violations	Cognition may follow interference-like principles	Theoretical; no identified neural substrate
Stochastic Schrödinger models [40]	Energy-based stochastic Schrödinger equation	Predicts spontaneous collapse → conserved mean energy	Framework linking micro-indeterminacy → macroscopic dynamics	Mathematical only; biological relevance undemonstrated
Quantum biology analogues (photosynthesis; magnetoreception; axonal photon models)	Spectroscopy → radical pair dynamics → photonic modelling	Long-lived coherence → entangled radical pairs → theoretical biphoton guidance	Demonstrates the feasibility of biological coherence	Neural evidence speculative; no direct verification in the brain
Analytical approaches (CRQA, MF-DFA)	Nonlinear EEG/MRI analyses	Recurrence & coherence-like patterns	Could reflect deterministic chaos → alternative to quantum mechanisms	Susceptible to false positives → requires robust null models

Take-home message: Across neuroimaging, behavioral, and theoretical domains, emerging studies increasingly report quantum-like or non-classical signatures in brain function. While not definitive evidence of quantum mechanisms, these findings provide testable models that extend beyond classical neuroscience and motivate rigorous empirical examination of whether non-classical processes contribute to perception, learning, and conscious experience. EEG, electroencephalography; ZQC, zero-quantum coherence; AIA, anomalous information anticipation; Q-coefficient, index for entanglement-structured variance; SSE, stochastic Schrödinger equation.

## Data Availability

No new data were created or analyzed in this study. Data sharing is not applicable to this article.

## Abbreviations

The following abbreviations are used in this manuscript:

ACT-R	Adaptive Control of Thought–Rationale
A-C	Access Consciousness
AIA	Anomalous Information Anticipation
ANN(s)	Artificial Neural Network(s)
BDNF	Brain-Derived Neurotrophic Factor
CRQA	Cross-Recurrence Quantification Analysis
EEG	Electroencephalography
FFA	Free Fatty Acids
fMRI	Functional Magnetic Resonance Imaging
GPT	Generalized Probability Theory
MEG	Magnetoencephalography
MF-DFA	Multifractal Detrended Fluctuation Analysis
MRI	Magnetic Resonance Imaging
MTL	Medial Temporal Lobe
NV	Nitrogen-Vacancy (diamond magnetometry)
Orch OR	Orchestrated Objective Reduction
P-C	Phenomenal Consciousness
Q (coefficient)	Quantum-Multilinear Integrated Coefficient
QAOA	Quantum Approximate Optimization Algorithm
QNN(s)	Quantum Neural Network(s)
SQC	Single Quantum Coherence
SP-RP	Stated Preference–Revealed Preference
SSE	Stochastic Schrödinger Equation
UtPBM	Unilateral Transcranial Photobiomodulation
ZQC	Zero Quantum Coherence

## References

[1] Chalmers D.J. (1995). Facing up to the problem of consciousness. J. Conscious. Stud..

[2] Keppler J. (2025). Macroscopic quantum effects in the brain: New insights into the fundamental principle underlying conscious processes. Front. Hum. Neurosci..

[3] Theise N.D., Tuszynski J.A. (2026). Non-linearity, complexity, and quantization concepts in biology. Front. Hum. Neurosci..

[4] Brody D.C. (2023). Quantum formalism for the dynamics of cognitive psychology. Sci. Rep..

[5] Gunji Y.-P., Sonoda K., Basios V. (2016). Quantum cognition based on an ambiguous representation derived from a rough set approximation. Biosystems.

[6] Khrennikov A., Ozawa M., Benninger F., Shor O. (2025). Coupling quantum-like cognition with the neuronal networks within generalized probability theory. J. Math. Psychol..

[7] Acacio de Barros J., Suppes P. (2009). Quantum mechanics, interference, and the brain. J. Math. Psychol..

[8] Bruza P.D., Wang Z., Busemeyer J.R. (2015). Quantum cognition: A new theoretical approach to psychology. Trends Cogn. Sci..

[9] Wang Z., Busemeyer J.R. (2013). A Quantum Question Order Model Supported by Empirical Tests of an A Priori and Precise Prediction. Top. Cogn. Sci..

[10] Tegmark M. (2000). Importance of quantum decoherence in brain processes. Phys. Rev. E.

[11] Rosa L.P., Faber J. (2004). Quantum models of the mind: Are they compatible with environment decoherence?. Phys. Rev. E.

[12] Lambert N., Chen Y.-N., Cheng Y.-C., Li C.-M., Chen G.-Y., Nori F. (2013). Quantum biology. Nat. Phys..

[13] Hameroff S., Penrose R. (2014). Consciousness in the universe: A review of the ‘Orch OR’ theory. Phys. Life Rev..

[14] Liu Z., Chen Y.-C., Ao P. (2024). Entangled biphoton generation in the myelin sheath. Phys. Rev. E.

[15] Zarkeshian P., Kergan T., Ghobadi R., Nicola W., Simon C. (2022). Photons guided by axons may enable backpropagation-based learning in the brain. Sci. Rep..

[16] Neven H., Zalcman A., Read P., Kosik K.S., van der Molen T., Bouwmeester D., Bodnia E., Turin L., Koch C. (2024). Testing the Conjecture That Quantum Processes Create Conscious Experience. Entropy.

[17] Kerskens C.M., López Pérez D. (2022). Experimental indications of non-classical brain functions. J. Phys. Commun..

[18] Jedlicka P. (2017). Revisiting the Quantum Brain Hypothesis: Toward Quantum (Neuro)biology?. Front. Mol. Neurosci..

[19] Escolà-Gascón Á. (2024). Our brains sense the future through a new quantum-like implicit learning mechanism. Brain Res. Bull..

[20] Escolà-Gascón Á. (2025). Evidence of quantum-entangled higher states of consciousness. Comput. Struct. Biotechnol. J..

[21] Escolà-Gascón Á., Benito-León J. (2025). Mathematical proof of the Fisher-Escolà Q statistical distribution in quantum consciousness modeling. Comput. Struct. Biotechnol. J..

[22] Gassab L., Pusuluk O., Cattaneo M., Müstecaplıoğlu Ö.E. (2025). Quantum Models of Consciousness from a Quantum Information Science Perspective. Entropy.

[23] Zhou M.-G., Liu Z.-P., Yin H.-L., Li C.-L., Xu T.-K., Chen Z.-B. (2023). Quantum Neural Network for Quantum Neural Computing. Research.

[24] Silberstein R.B., Bigelow F.J. (2024). Brain functional connectivity correlates of anomalous interaction between sensorily isolated monozygotic twins. Front. Hum. Neurosci..

[25] Abramov D.M., Quintanilha D.d.F., Lima H.S., Costa R.P., Kamil-Leite C., Lazarev V.V., Tsallis C. (2025). Neurophysiological correlates to the human brain complexity through q-statistical analysis of electroencephalogram. Sci. Rep..

[26] Georgiev D.D. (2025). Quantum information theoretic approach to the hard problem of consciousness. Biosystems.

[27] Schiffer F. (2025). A four-field quantum model of life, subjectivity, consciousness, and memory: Integrating dual-brain psychology and biophoton quantum interactions. Med. Hypotheses.

[28] Marshall P. (2023). The role of quantum mechanics in cognition-based evolution. Prog. Biophys. Mol. Biol..

[29] Yu J.G., Jayakrishnan R. (2018). A quantum cognition model for bridging stated and revealed preference. Transp. Res. Part B Methodol..

[30] Pathak P., Innan N., Marchisio A., Shafique M., Cheng L., Saurabh N., Mao Y. (2026). Quantum-enhanced decision-making in ACT-R: Cognitive architectures, decision-making, and quantum optimization. Quantum Computational AI.

[31] Swan M., dos Santos R.P., Witte F. (2022). Quantum Neurobiology. Quantum Rep..

[32] Swan M., dos Santos R.P., Lebedev M., Witte F. (2022). Quantum Computing for the Brain.

[33] Aerts D., Broekaert J., Gabora L. (2011). A case for applying an abstracted quantum formalism to cognition. New Ideas Psychol..

[34] Levine Y., Sharir O., Cohen N., Shashua A. (2019). Quantum Entanglement in Deep Learning Architectures. Phys. Rev. Lett..

[35] Kyriazos T., Poga M. (2024). Quantum concepts in Psychology: Exploring the interplay of physics and the human psyche. Biosystems.

[36] Gunji Y.-P., Shinohara S., Haruna T., Basios V. (2017). Inverse Bayesian inference as a key of consciousness featuring a macroscopic quantum logical structure. Biosystems.

[37] Eeles E., Pourzinal D., Baland J., Ray J. (2025). Schrödinger’s cat and mouse: An adapted thought experiment for the context of consciousness. Behav. Brain Res..

[38] Wiest M.C., Puniani A.S. (2025). Conscious active inference I: A quantum model naturally implements the path integral needed for real-time planning and control. Comput. Struct. Biotechnol. J..

[39] Wiest M.C., Puniani A.S. (2025). Conscious active inference II: Quantum orchestrated objective reduction among intraneuronal microtubules naturally accounts for discrete perceptual cycles. Comput. Struct. Biotechnol. J..

[40] Brody D.C., Hughston L.P. (2006). Quantum noise and stochastic reduction. J. Phys. A Math. Gen..

[41] Waddup O.J., Yearsley J.M., Blasiak P., Pothos E.M. (2023). Temporal Bell inequalities in cognition. Psychon. Bull. Rev..

[42] Hameroff S., Penrose R. (1996). Orchestrated reduction of quantum coherence in brain microtubules: A model for consciousness. Math. Comput. Simul..

[43] Vitas M. (2025). Towards a Possible Definition of Consciousness. Biosystems.

[44] Bardella G., Franchini S., Pani P., Ferraina S. (2024). Lattice physics approaches for neural networks. iScience.

[45] Khrennikov A. (2015). Quantum-like modeling of cognition. Front. Phys..

[46] Pothos E.M., Yearsley J.M., Plotnitsky A., Haven E. (2023). Quantum Cognition: Quo Vadis?. The Quantum-Like Revolution: A Festschrift for Andrei Khrennikov.

[47] Khrennikov A., Basieva I., Pothos E.M., Yamato I. (2018). Quantum probability in decision making from quantum information representation of neuronal states. Sci. Rep..

[48] Baars B.J., Edelman D.B. (2012). Consciousness, biology and quantum hypotheses. Phys. Life Rev..

[49] Loboguerrero D.A. (2025). The collapse of the wave function as the mediator of free will in prime neurons. Front. Neurosci..

[50] Wiest M.C. (2025). A quantum microtubule substrate of consciousness is experimentally supported and solves the binding and epiphenomenalism problems. Neurosci. Conscious..

[51] Freitas da Rocha A., Pereira A., Bezerra Coutinho F.A. (2001). N-methyl-d-aspartate channel and consciousness: From signal coincidence detection to quantum computing. Prog. Neurobiol..

[52] Pereira A., Poznański R.R., Brändas E.J. (2020). Classical-quantum interfaces in living neural tissue supporting conscious functions. Advances in Quantum Chemistry.

